# Efficacy of a Synergistic Blend of Organic Acids and ß-1,4 Mannobiose on Cecal *Salmonella* Counts and Growth Performance in *Salmonella* Challenged Broiler Chickens: A Meta-Analysis

**DOI:** 10.3390/ani11102988

**Published:** 2021-10-17

**Authors:** Sandra J. A. van Kuijk, Yanming Han

**Affiliations:** Trouw Nutrition R&D, Stationsstraat 77, 3811 MH Amersfoort, The Netherlands

**Keywords:** broiler chicken, growth performance, mannobiose, meta-analysis, organic acids, *Salmonella*

## Abstract

**Simple Summary:**

*Salmonella* may cause serious diarrhea in consumers of poultry products. Control of *Salmonella* in poultry with antibiotics has caused antimicrobial resistance issues. *Salmonella* can colonize the gut of chickens, meaning that an in-feed solution may prevent infection. A synergistic blend containing organic acids and ß-1,4 mannobiose as main ingredients were developed to reach different parts of the gut. It was hypothesized that this synergistic blend decreases *Salmonella* contamination of chickens. Several, non-published, studies have been performed to test the effect of this synergistic blend in chickens infected with *Salmonella*. The results of these studies were combined into a large meta-analysis to draw conclusions regardless of study design and geographical location. This state-of-the-art statistical method did show that feeding the synergistic blend to chickens could decrease *Salmonella* in comparison to a control diet. This decrease was most clear during the first 14 days after initiation of the *Salmonella* infection. In addition, the birds did grow more efficiently when the synergistic blend was fed.

**Abstract:**

This study aimed at investigating the effect of a functional synergistic feed additive blend, containing organic acids and ß-1,4 mannobiose, on cecal *Salmonella* counts and growth performance of broiler chickens. A meta-analysis combining 13 individual studies, executed in *Salmonella*-challenged broilers comparing a control diet with and without the synergistic blend, was performed. Cecal *Salmonella* colonies and overall growth performance were measured. Raw data from all studies were combined and analyzed using PROC MIXED in SAS, taking the within and between-study variation into account. In the first 14 days post-inoculation (DPI), cecal *Salmonella* was reduced by 0.429 log CFU/g (*p* = 0.011, *n* = 10 comparisons from five studies) when feeding the synergistic blend compared to the control group. During 15–34 DPI, the overall means were not different between treatments (0.069 log CFU/g; *p* = 0.519, *n* = 12 comparisons from eight studies). The feed conversion ratio was improved when feeding the synergistic blend compared to the control diet (1.474 vs. 1.482, respectively; *p* = 0.002). In conclusion, feeding a synergistic blend, containing organic acids and ß-1,4 mannobiose amongst other ingredients, reduced cecal *Salmonella* counts during the first 14 DPI and improved growth performance of *Salmonella* challenged broilers compared to a control diet.

## 1. Introduction

*Salmonella* is a major foodborne pathogen that not only results in direct food safety issues by causing diarrhea and other health problems in humans; indirectly, potentially more severe problems can occur, as several *Salmonella* serovars are described to be resistant to one or more antibiotics. In a recent overview [1], the median prevalence values of *Salmonella* in broiler chickens, raw chicken meat, and in eggs and egg-laying hens were 40.5%, 30%, and 40%, respectively. Antimicrobial therapy is the first choice of treatment for this bacterial infection; however, antimicrobial resistance has become a problem due to the misuse of antibiotics, both in human medicine and animal production [1]. In the period from 2000–2004, 15% of the clinical *Salmonella* isolates in Europe were identified as multi-drug resistant, and this number was even up to 84% of the clinical isolates in the USA in 2005–2006 [2]. More recently, published in 2020 summarizing 45 scientific publications showing an incidence of multi-drug resistant *Salmonella* in 91.1% of the poultry farms across the world [1]. Between 15 and 35% of the *Salmonella* related foodborne illnesses are related to poultry meat Hoffman et al. [3]. The spread of *Salmonella* in the poultry production chain is multifactorial and linked to feed, drinking water, the hatchery, pests, wild birds, insects, infected dead or alive birds, litter, personnel, equipment and vehicles [4,5,6,7,8]. Since feed can be a significant source for *Salmonella* contamination [4], an in-feed solution is postulated as one of the key steps in mitigating *Salmonella* contamination. On top of other measures, in-feed solutions focusing on strengthening natural barriers in the intestinal tract can be effective in reducing the infection and prevalence of *Salmonella* on farms. 

Several in-feed solutions have been described in the literature. Infection can be prevented via lowering the pH of the gastrointestinal tract, for example via organic acids, specifically (encapsulated) short chain fatty acids (SCFA) [9,10,11]. Whereas SCFA act mostly in the upper gastrointestinal tract, medium chain fatty acids (MCFA) having a greater antibacterial activity against *Salmonella* are released later in the gastrointestinal tract [5]. However, to reach the lowest parts of the gastrointestinal tract, MCFA need encapsulation [5,10,12]. The coated form of butyric acid has been described to be effective in *Salmonella* control in the cecum of broiler chickens challenged with *Salmonella* Enteritidis, indicating that the coating of butyrate results in effects in the lower gastrointestinal tract [12]. Sodium butyrate was shown to improve the villi and mucosa of the intestines and in general has a beneficial effect on gut microbiota [13], general gut health and growth performance when compared to a negative control or antibiotic growth promotors [14]. Hydrolyzed copra meal is an ingredient containing significant amounts of ß-1,4 mannobiose, a compound described to have an effect on *Salmonella* by increasing phagocytosis by macrophages in vitro [15] and reducing *Salmonella* in the cecum of challenged broiler chickens in vivo [16]. Because of the multifactorial nature of *Salmonella* infections and differences in modes of action of the aforementioned ingredients, a blend of different feed ingredients may have synergistic effects in targeting *Salmonella*. In addition, the complex nature of *Salmonella* infections often causes variable results in scientific experiments, especially when these are done under different circumstances. The objective of the present study was to investigate the overall effect of a synergistic feed additive blend including ß-1,4 mannobiose, SCFA’s, MCFA’s, and microencapsulated butyrate on *Salmonella* counts in the cecum of *Salmonella* challenged broiler chickens, regardless of experimental conditions. Therefore, several experiments performed under different circumstances were combined into one meta-analysis. It is hypothesized that fewer *Salmonella* would colonize the cecum in birds fed the synergistic blend compared to the control birds. In addition, it is hypothesized that the synergistic concept not only affects *Salmonella* counts in the cecum but also improves the growth performance of *Salmonella*-challenged broiler chickens. 

## 2. Materials and Methods

All animal procedures in the different studies were approved by the local animal committees according to the standard procedures of the universities/institutes, where the studies were performed. 

### 2.1. Selection of Studies

The following inclusion criteria were maintained: (1) studies must have been done in broiler chickens starting from day-old; (2) broiler chickens must have been challenged at any age with any serovar of *Salmonella* except for pathogenic serovars for poultry; (3) *Salmonella*-challenged birds fed the synergistic blend must be compared to *Salmonella*-challenged birds fed a non-supplemented control diet; (4) the raw data must be available for the analysis. Studies were excluded from the meta-analysis when: (1) the inclusion criteria were not fulfilled; (2) data of the study were regarded as unreliable due to (unforeseen) circumstances; (3) *Salmonella* counts were lower than 3 log CFU/g cecal digesta during the first 20 days of the study indicating a failed challenge model. All considered studies were performed at Trouw Nutrition, or with collaborations of Trouw Nutrition. None of the studies were published.

### 2.2. Study Design

All studies were done with broiler chickens starting from one day old. In all studies, birds received a *Salmonella* challenge but the age at initiation of the challenge differed per study. The age at introduction of *Salmonella* challenge varied between day 1 and 10 and is summarized in Table 1 for each individual study. The serovars used in the different studies are summarized in Table 1, all were isolated from the field. Two types of challenges were used (Table 1): (1) An infection model, where all birds in a pen were inoculated with the same amount of inoculum; (2) A seeder model, where a selected number of birds per pen were inoculated with the respective *Salmonella* serovar and placed in the pen to spread the infection to other birds. In all cases, the dietary treatments started on day 1 of the study, which was well before the *Salmonella* challenge was introduced. The dietary treatments consisted of either a control diet, formulated to meet or exceed the requirements set by National Research Council [17] following local practices, or the control diet including the synergistic blend (Fysal Fit-4, Trouw Nutrition, the Netherlands). The same blend was used in all individual studies and was a proprietary mixture of SCFA, MCFA, hydrolyzed copra meal (containing 13% ß-1,4 mannobiose) and microencapsulated sodium salts of butyric acid. The synergistic blend was included at 3 kg/t in the first feeding phase of all studies, up to a maximum of 23 days of the study, and then lowered to a minimum of 1 kg/t until the end of each study. 

### 2.3. Parameters

The meta-analysis included two major parts, one being the *Salmonella* data and the other one the growth performance for the overall study period. For the *Salmonella* assessment, a minimum of one, up to 10 birds per pen, dependent on the study design, were selected for the sampling of cecal digesta at different days post-inoculation. In studies 1, 5, 6, 8 and 9, the complete content of one cecum per bird was used, while in the other studies (3, 4, 7, 10, 11, 12, 13) the complete content of both ceca was used for *Salmonella* enumeration. In the case of a seeder model, seeder birds were excluded from the analysis. *Salmonella* counts were based on plating methods using Brilliant Green Agar with supplements (origin unknown) incubated at 37 °C for 24 h (studies 01, 03, 05, 07, 08, 09 and 10 in Table 1), Bismuth Silfite Agar with supplemented Novobiocin and *Salmonella* growth supplement (origin unknown) incubated at 37 °C for 24 h (study 02 in Table 1), or xylose lysine deoxycholate (XLD) agar containing nalidixic acid at 50 µg/mL (origin unknown) incubated at 42 °C for 24 h (study 13 on Table 1). *Salmonella* counts below the detection limit were regarded as negative (given a value of 0). *Salmonella* counts, as average per pen, were expressed as log colony forming units (CFU)/g cecal digesta.

Bodyweight was measured per pen at the start and end of each study. The feed intake was measured per pen for the overall study period. Growth performance was calculated for the total study period as average daily gain (ADG) in g/bird/day, average daily feed intake (ADFI) in g/bird/day and feed:gain ratio as feed conversion ratio (FCR) in g:g. 

### 2.4. Statistics

All raw data were combined into one Excel file. Individual pens in each study were considered as the experimental unit. Statistical analysis was performed using the SAS software, version 9.4 (SAS Institute Inc., Cary, NC, USA). All parameters were compared between infected birds fed the synergistic blend and infected birds fed a control diet. *Salmonella* counts were split into two different data sets, one including counts between 0- and 14-days post-inoculation (DPI) and another including counts between 15 and 34 DPI. All parameters were analyzed using the MIXED procedure in SAS. In each model the within-study variation and between-study variation were included in the random and repeated statement, respectively, not assuming equal variances of treatments. Results were regarded as significant at *p* < 0.05. 

## 3. Results

### 3.1. Selection of Studies

Based on the inclusion and exclusion criteria as defined in the materials and methods, 13 out of 16 studies were selected for the meta-analysis. The first study excluded was because *Salmonella* Pullorum was used in the challenge model. The second study excluded was because the minimum infection level of 3 log CFU/g *Salmonella* in the cecum was not reached within the first 20 days of the study. The third study excluded showed results that cannot be explained by any biological processes. In this latter study, instead of an expected steady decline of *Salmonella* counts, similar to the natural course of a *Salmonella* infection, a sudden sharp increase in *Salmonella* counts was observed. The reason for the unexpected observation in this study is unknown, therefore this study was removed from the meta-analysis. 

Due to differences in the study set-ups, not all 13 selected studies could be included in the analysis for all parameters. Differences in timings of measurements, measurement methods (quantitative vs. qualitative *Salmonella* identification) and availability of growth performance data resulted in a different number of studies included for the individual parameters. Table 1 shows the included studies with their key characteristics and parameters.

### 3.2. Cecal Salmonella Counts

**Figure 1:**
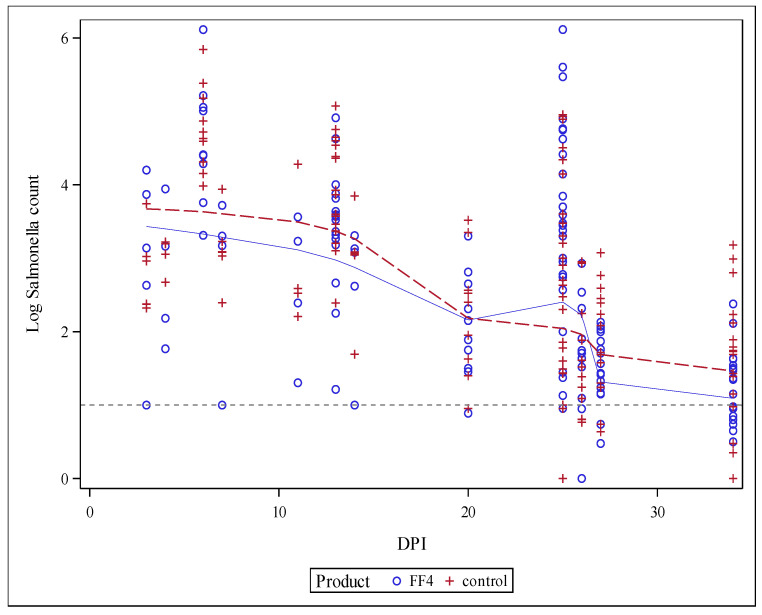
Individual *Salmonella* counts (log CFU/g) on different days post-inoculation (DPI). Each plus or circle represents one measurement per pen of the control or synergistic blend group respectively. The solid blue line is fitted through the data of the synergistic blend group, the dotted red line is fitted through the data of the control group. The vertical line represents where the split was made in the data set at 14 DPI.

Figure 1 shows an overview of the cecal counts for the different DPI in all studies. It shows that the peak in *Salmonella* counts in the cecum appeared to happen during the first 14 DPI. For this reason, the meta-analysis has been grouped based on DPI with the first group being 0–14 DPI and the second from 15–34 DPI. 

**Figure 2:**
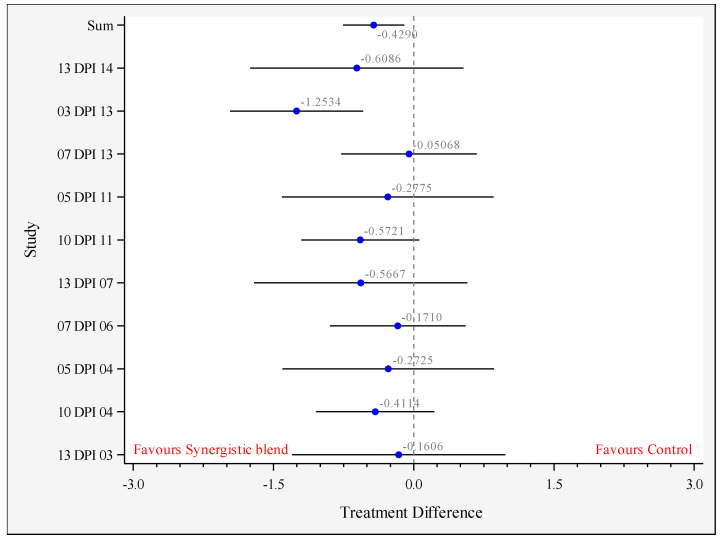
Forest plot showing the difference of the mean between control and synergistic blend (Treatment) for each study, and all studies combined (sum; *p* = 0.011), 0–14 days post-inoculation (DPI). The circles represent the log CFU/g difference between treatments, the bars represent the 95% confidence limits and the vertical line at 0 represents the value of no difference between treatments. Legend: XX DPI YY with XX = study number (see Table 1) and YY = days post-inoculation.

Five studies could be included in the analysis 0–14 DPI, due to the study designs. Figure 2 and Table 2 show the results of the meta-analysis 0–14 DPI. Figure 2 shows the mean difference in log CFU *Salmonella* counts in the cecum per study per DPI. The difference between the overall means of all studies is 0.429 log CFU/g (*p* = 0.011, *n* = 10 comparisons from five studies) in favor of the synergistic blend. 

**Figure 3:**
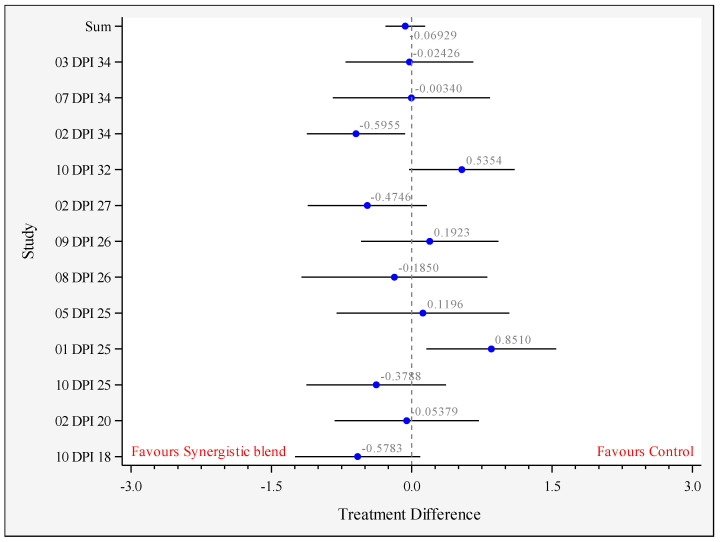
Forest plot showing the difference of the mean between control and synergistic blend (Treatment) for each study, and all studies combined (sum; *p* = 0.591), between 15–34 days post-inoculation (DPI). The circles represent the log CFU/g difference between treatments, the bars represent the 95% confidence limits and the vertical line at 0 represents the value of no difference between treatments. Legend: XX DPI YY with XX = study number (see Table 1) and YY = days post-inoculation.

Eight studies were included in the analysis of cecal *Salmonella* counts for 15–34 DPI. The results of this analysis are summarized in Figure 3 and Table 2. The overall means were not significantly different (0.069 log CFU/g; *p* = 0.519, *n* = 12 comparisons from eight studies) between the treatments. 

### 3.3. Growth Performance

**Figure 4:**
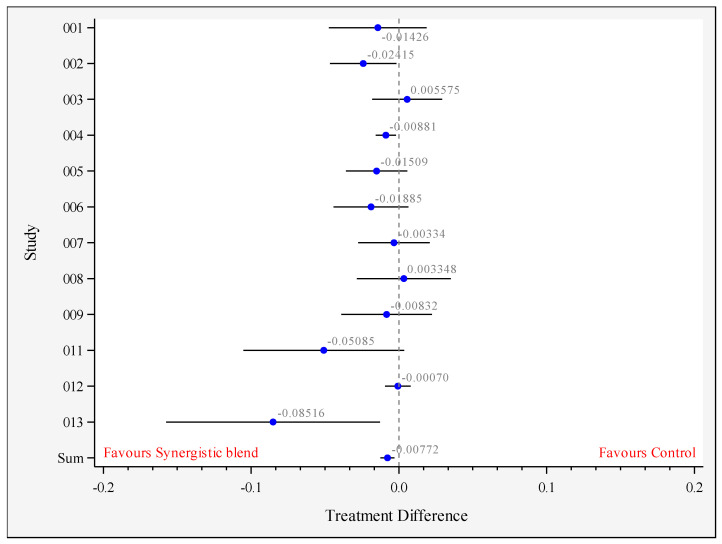
Forest plot for a feed conversion ratio of the overall study period between control and synergistic blend (Treatment). The circles represent the log CFU/g difference between treatments, the bars represent the 95% confidence limits and the vertical line at 0 represents the value of no difference between treatments. Legend: 0XX with XX = study number (See Table 1).

The growth performance results are summarized in Table 3. When including all studies as indicated in Table 1, there were no significant differences in final body weight, ADG and ADFI. The feed conversion ratio was significantly improved in the birds fed the synergistic blend compared to the control diet (1.474 vs. 1.482 respectively; *p* = 0.002, *n* = 12 comparisons, Figure 4).

In line with the *Salmonella* count analysis, where not all studies could be included in each analysis, an additional analysis for growth performance was performed. Taking into account only the same five studies (study number 3, 5, 7, 10, 13) used for the analysis on the *Salmonella* counts 0–14 DPI, a tendency towards an increased body weight of 21.8 g compared to the control diet (2317.7 g vs. 2295.0 g; *p* = 0.097, *n* = 5 comparisons) was observed for the chickens fed the synergistic feed additive blend, while the beneficial effect on FCR was not observed here. 

## 4. Discussion

In total 13 studies were selected for inclusion in the meta-analysis. One study was excluded because of the use of *Salmonella* Pullorum. This serovar is known to be pathogenic for broiler chickens [6,18]. *Salmonella* Pullorum does cause disease in poultry, while the other serovars used in the current study will not cause any clinical signs in infected birds [18]. The pathogenicity of *Salmonella* Pullorum might influence infection and growth performance differently, compared to other serovars. Another study was excluded because it did not reach the minimum of 3 log CFU/g cecal *Salmonella* counts. As the *Salmonella* detection methods used here have a detection limit between 1 and 2 log CFU/g, it is expected that any small reduction in birds having cecal counts below 3 log CFU/g would result in undetectable or highly variable results.

All data combined as shown in Figure 1 indicated a peak in cecal *Salmonella* counts in the first 14 days post-inoculation, which is similar to the trends described by Stern [19] for birds at 4 days of age infected with three different *Salmonella* serovars. This decrease in *Salmonella* counts over time was attributed to the maturation of the intestinal flora of young chicks [19]. In the current study, the most significant differences were observed in the first 14 days post-inoculation. The lack of difference in data thereafter is likely related to an adaptive decrease in *Salmonella* colonization in growing chicks [19]. The current results confirm the natural course of *Salmonella* infection and show that interventions should be applied within the first 2 weeks when inoculation occurs at a younger age. 

The serovars used in the current meta-analysis, *Salmonella* Enteritidis, *Salmonella* Typhimurium or *Salmonella* Heidelberg, represent the most common ones in Europe and the USA [5,7,20,21]. Not only is a food safety risk associated with these serovars, some, such as *Salmonella* Typhimurium, are described to have antibiotic resistance [2,22]. In commercial practice, more different serovars are being detected, such as *Salmonella* Infantis that is often related to antimicrobial resistance [8]. In this study, not enough data was available to draw specific conclusions per serovar. Therefore, future research should also look at the effect of different *Salmonella* control measures on each different serovar and more serovars should be studied. Next to different serovars, the studies included were different in regard to the challenge models as well as regional differences in animals and diets. None of these differences were considered in the statistical analysis due to insufficient data. However, the way the current analysis was executed indicates that the synergistic blend significantly reduces cecal *Salmonella* counts regardless of the study conditions. 

The synergistic blend used in the current study is a mixture of active ingredients including SCFA, MCFA, hydrolyzed copra meal, sodium salts of butyric acid. The observed beneficial effects on *Salmonella* counts and growth performance cannot be attributed to specific single ingredients but is most likely the combination of the different modes of action of these ingredients. SCFA and MCFA are described to be effective against *Salmonella* [5,9,23]. Although these compounds may have an effect on *Salmonella* in feed already, or have a slight effect on the crop pH, the most effect is to be expected if they manage to reach the lower part of the digestive tract, as this is the main colonization site of *Salmonella* [23]. In addition, there is a large body of evidence on SCFA and MCFA maintaining the desired performance in the absence of antibiotics. In their literature review, Polycarpo et al. [24] report up to 5.67% improvement in FCR in microbiologically challenged broiler chickens fed these organic acids.

The ß-1,4 mannobiose originating from hydrolyzed copra meal as used in the synergistic blend has been linked to *Salmonella* reduction in the cecum of broiler chickens challenged with *Salmonella* at two weeks of age [16]. The same ingredient is shown to improve immune response, by increased IgA levels in feces and upregulated immune-related genes in the ileum, in non-challenged broiler chickens [25]. In addition, in vitro data show increased phagocytosis by macrophages after exposure to ß-1,4 mannobiose [15]. In line with the current study, ß-1,4 mannobiose did improve final body weight and feed efficiency [26]. In another study, Ibuki et al. [27] showed an improved body weight (+36.3 g), breast meat (+12.4 g) and thigh weights (+9.3 g) at 22 days of age in broiler chickens fed ß-1,4 mannobiose. In contrast, Ibuki et al. [25] observed no significant effects of this ingredient on growth performance.

Another ingredient in the blend, butyric acid has been described to reduce fecal shedding of *Salmonella* Enteridis when administered in protected coated form or in the form of impregnated microbeads [12,28,29]. Sodium butyrate improves gut health by improving the gut wall and mucosa and improving the microbial balance [13]. Under non-challenged conditions, butyric acid supplemented at 0.4% or 0.6% improved final body weight and FCR to the same extent or even numerically higher than the antibiotic treatment in comparison with the negative control [14]. The FCR improvement (0.52%) by the synergistic blend as observed in the current study is smaller compared to literature describing single ingredients. In line with the current study, the results described by Aljumaah et al. [30], showed a numerically improved FCR in *Salmonella* Typhimurium infected chickens when fed the synergistic blend, compared to the infected control birds (1.554 vs. 1.646, respectively). It is important to note that the difference in effect on growth performance between the literature and the current study may be related to the type and inclusion level of organic acids used in the synergistic blend, but also to the study designs having the main focus on *Salmonella* reduction in the current study.

The current synergistic blend did reduce the cecal counts of *Salmonella* significantly, however it did not completely eliminate *Salmonella* in the cecum. This is in line with Abudabos et al. [31], who observed a numerical reduction from 4.38 to 1.83 log CFU/g cecal *Salmonella* (Typhimurium) when birds were fed a blend of organic acids similar to that used in the current study compared to the infected control birds. In contrast to the cecum, Abudabos et al. [31] describe a complete elimination of *Salmonella* in the ileum when feeding the organic acid blend. It may be argued that complete elimination of *Salmonella* was not expected, due to the relatively high number of *Salmonella* in the inoculum used to get a successful model.

## 5. Conclusions

The current study shows that supplementing a synergistic blend containing a mixture of SCFA, MCFA, hydrolyzed copra meal (containing ß-1,4 mannobiose) and microencapsulated sodium salts of butyric acid as main ingredients, significantly reduced cecal *Salmonella* counts within the first 14 days post-inoculation compared to a control diet. The variety in study designs included in the meta-analysis indicates that the synergistic blend is effective under a wide variety of conditions. In addition, the synergistic blend tended to improve FCR over the entire growth period.

## Figures and Tables

**Table 1:** Studies included in the meta-analysis.

Study Number	Facility	Country	Study Duration (Days)	Model	Salmonella Serovar	Day of Inoculation	Included Parameters ^1^
01	Trouw Nutrition Poultry Research Centre	Spain	32	Infection	Enteritidis	7	Growth performance, 15–34 DPI
02	Trouw Nutrition Poultry Research Centre	Spain	42	Infection	Typhimurium	8	Growth performance, 15–34 DPI
03	Trouw Nutrition Poultry Research Centre	Spain	35	Infection	Enteritidis	8	Growth performance, 0–14 DPI, 15–34 DPI
04	Trouw Nutrition Poultry Research Centre	Spain	42	Seeder	Enteritidis	8	Growth performance
05	Trouw Nutrition Poultry Research Centre	Spain	32	Infection	Enteritidis	7	Growth performance, 0–14 DPI, 15–34 DPI
06	Trouw Nutrition Poultry Research Centre	Spain	39	Seeder	Enteritidis	7	Growth performance
07	Trouw Nutrition Poultry Research Centre	Spain	42	Infection	Enteritidis	8	Growth performance, 0–14 DPI, 15–34 DPI
08	Trouw Nutrition Poultry Research Centre	Spain	33	Infection	Enteritidis	7	Growth performance, 15–34 DPI
09	Trouw Nutrition Poultry Research Centre	Spain	33	Infection	Typhimurium	7	Growth performance, 15–34 DPI
10	Mercolab	Brazil	35	Seeder	Heidelberg	3	0–14 DPI, 15–34 DPI
11	USP	Brazil	42	Infection	Heidelberg	10	Growth performance
12	IRTA	Spain	35	Seeder	Enteritidis	1	Growth performance
13	Chulalongkorn	Thailand	35	Infection	Typhimurium	7	Growth performance, 0–14 DPI

^1^ Performance = growth performance, 0–14 DPI = *Salmonella* counts between days 0 and 14 post-inoculation, 15–34 DPI = *Salmonella* counts between days 15 and 35 post-inoculation.

**Table 2:** Meta-analysis of cecal *Salmonella* counts.

Outcome	*Salmonella* 0–14 DPI ^1^ (Log CFU/g)	*Salmonella* 15–34 DPI ^2^ (Log CFU/g)
Mean-Control	3.097 ^a^	1.605
Mean-Synergistic blend	2.669 ^b^	1.536
Difference of the means	−0.429	−0.069
Standard error of the difference	0.164	0.107
Lower 95% confidence limit	−0.757	−0.281
Upper 95% confidence limit	−0.101	0.142
*p*-value	0.011	0.519

^1^ 0–14 DPI = *Salmonella* counts between days 0 and 14 post-inoculation, ^2^ 15–34 DPI = *Salmonella* counts between days 15 and 35 post-inoculation. ^a,b^ different superscripts within a column indicate significant difference between means (*p* ≤ 0.05).

**Table 3:** Results of the meta-analysis on growth performance for the overall study period.

Outcome	Final BW ^1^ (g)	ADG ^2^ (g/Bird/Day)	ADFI ^3^ (g/Bird/Day)	FCR (g:g) ^4^
Mean-Control	2304.5	62.31	92.36	1.482 ^a^
Mean-Synergistic blend	2304.8	62.50	91.97	1.474 ^b^
Difference between means	0.28	0.188	−0.386	−0.0077
Standard error of the difference	18.64	0.291	0.396	0.0023
Lower 95% confidence limit	−36.45	−0.388	−1.179	−0.0124
Upper 95% confidence limit	37.01	0.764	0.407	−0.0030
*p*-value	0.988	0.518	0.334	0.002

^1^ BW = body weight, ^2^ ADG = average daily gain, ^3^ ADFI = average daily feed intake, ^4^ FCR = feed conversion ratio, ^a,b^ different superscripts within a column indicate a significant difference between means (*p* ≤ 0.05).

## Data Availability

The data presented in this study are available on request from the corresponding author. The data are not publicly available due to confidentiality between the contributed parties.
